# Kinetics of Bilirubin and Ammonia Elimination during Hemadsorption Therapy in Secondary Sclerosing Cholangitis Following ECMO Therapy and Severe COVID-19

**DOI:** 10.3390/biomedicines9121841

**Published:** 2021-12-05

**Authors:** Désirée Tampe, Peter Korsten, Sebastian C. B. Bremer, Martin S. Winkler, Björn Tampe

**Affiliations:** 1 Department of Nephrology and Rheumatology, University Medical Center Göttingen, 37075 Göttingen, Germany; desiree.tampe@med.uni-goettingen.de (D.T.); peter.korsten@med.uni-goettingen.de (P.K.); 2 Department of Gastroenterology, Gastrointestinal Oncology and Endocrinology, University Medical Center Göttingen, 37075 Göttingen, Germany; sebastian.bremer@med.uni-goettingen.de; 3 Department of Anesthesiology, Emergency and Intensive Care Medicine, University Medical Center Göttingen, 37075 Göttingen, Germany; martin.winkler@med.uni-goettingen.de

**Keywords:** bilirubin elimination, ammonia elimination, hemadsorption, CytoSorb, secondary sclerosing cholangitis, SSC-CIP, COVID-19, extracorporeal membrane oxygenation, intensive care medicine

## Abstract

In critically ill patients, liver dysfunction often results in coagulopathy and encephalopathy and is associated with high mortality. Extracorporeal clearance of hepatotoxic metabolites, including bilirubin and ammonia, aims to attenuate further hepatocyte damage and liver injury, resulting in decreased mortality. The efficacy of hemadsorption combined with conventional hemodialysis to eliminate bilirubin and ammonia to support the liver’s excretory function in acute liver injury has been described previously. However, the optimal use of liver support systems in chronic liver dysfunction due to secondary sclerosing cholangitis in critically ill patients (SSC-CIP) has not been defined yet. We herein describe the kinetics of successful bilirubin and ammonia elimination by hemadsorption in a patient with SSC-CIP after extracorporeal membrane oxygenation (ECMO) therapy for severe acute respiratory distress syndrome (ARDS) in a patient with coronavirus disease 2019 (COVID-19). During the course of the disease, the patient developed laboratory signs of liver injury during ECMO therapy before clinically detectable jaundice or elevated bilirubin levels. A diagnosis of SSC-CIP was confirmed by endoscopic retrograde cholangiopancreatography (ERCP) based on intraductal filling defects in the intrahepatic bile ducts due to biliary casts. The patient showed stable elevations of bilirubin and ammonia levels thereafter, but presented with progressive nausea, vomiting, weakness, and exhaustion. Based on these laboratory findings, hemadsorption was combined with hemodialysis treatment and successfully eliminated bilirubin and ammonia. Moreover, direct comparison revealed that ammonia is more efficiently eliminated by hemadsorption than bilirubin levels. Clinical symptoms of nausea, vomiting, weakness, and exhaustion improved. In summary, bilirubin and ammonia were successfully eliminated by hemadsorption combined with hemodialysis treatment in SSC-CIP following ECMO therapy and severe COVID-19. This observation is particularly relevant since it has been reported that a considerable subset of critically ill patients with COVID-19 suffer from liver dysfunction associated with high mortality.

## 1. Introduction

In critically ill patients, liver dysfunction potentially results in coagulopathy and encephalopathy and is associated with high mortality [1,2]. Liver dysfunction can either occur because of primary liver diseases or result from secondary causes. Reasons for primary liver dysfunction are hepatotoxic agents, including drugs or viral infection, leading to acute liver dysfunction and potential recovery from injury [3,4]. More frequently, liver dysfunction is observed secondary to cholestasis, hypoxic liver injury, sepsis, or cardiogenic shock with acute and possibly persistent liver dysfunction in critically ill patients [5,6]. Among them, secondary sclerosing cholangitis (SSC) has been observed after hypoxic liver injury, but also in critically ill patients (SSC-CIP) requiring extracorporeal membrane oxygenation (ECMO) therapy [7]. SSC-CIP and impaired liver clearance cause accumulation of toxic metabolites (including elevated levels of bilirubin and ammonia) with sustained synthetic liver function [7]. In critically ill patients, elevated bilirubin levels are associated with higher mortality [8,9].

Furthermore, accumulation of ammonia can cause cerebral edema with a risk of persistent cerebral injury [10]. Treatment regimens of liver dysfunction in critically ill patients include prevention of ongoing liver injury, ultimately leading to the requirement for liver transplantation or death [11,12]. In addition, liver support systems are used as supportive therapy. Extracorporeal clearance of hepatotoxic metabolites, including bilirubin and ammonia, aims to attenuate further hepatocyte damage and liver injury, potentially resulting in decreased mortality [13]. Liver support systems include therapeutic plasma exchange, which has been shown to improve outcomes but may lead to hypotension and increased bleeding risk [14,15,16,17]. Another approach is albumin dialysis, as described with molecular adsorbent recirculating system (MARS) or advanced organ support (ADVOS) [18,19,20]. ADVOS is an advanced hemodialysis system combining organ support for the liver and kidneys. Although case studies have shown the efficacy of the ADVOS system in critically ill patients with acute liver dysfunction, it has not yet been established in daily intensive care practice [21,22]. Finally, the efficacy of hemadsorption in combination with conventional hemodialysis to eliminate bilirubin and ammonia to support the liver’s excretory function in acute liver injury has been described previously [23,24,25,26]. 

However, the role of liver support systems in chronic liver dysfunction, including SSC, has not yet been defined. We herein describe the kinetics of successful bilirubin and ammonia elimination by hemadsorption in a patient with SSC-CIP following ECMO therapy and coronavirus disease 2019 (COVID-19).

## 2. Case Description

**Figure 1:**
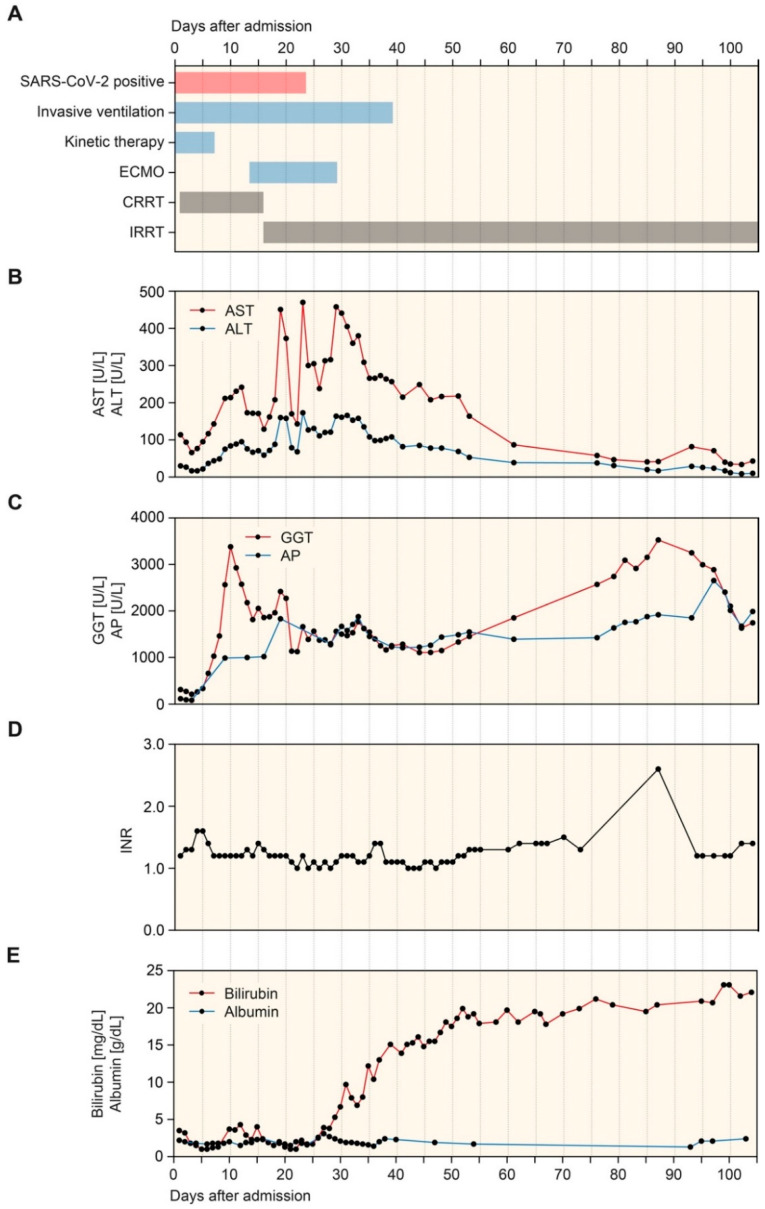
Timeline of COVID-19 disease course. (**A**) Timeline of treatment regimens after admission, kinetic therapy included prone positioning for at least 12 h per day. (**B**–**E**) Time course of plasma AST, ALT, GGT, AP, INR, bilirubin, and albumin. Abbreviations: ALT, alanine transaminase; AP, alkaline phosphatase; AST, aspartate transaminase; COVID-19, coronavirus disease 2019; GGT, gamma-glutamyl transferase; INR, international normalized ratio.

A 61-year-old woman with confirmed COVID-19 was admitted to a different hospital with a productive cough experienced for a few days. She required invasive assisted ventilation shortly after admission. Her medical history included rheumatoid arthritis, obstructive sleep apnea, and arterial hypertension. Because of progressive severe acute respiratory distress syndrome (ARDS), the patient was transferred to our tertiary care hospital for kinetic therapy (prone positioning for at least 12 h per day) and continuous renal replacement therapy (CRRT) due to oliguric acute kidney injury (AKI) (Figure 1A). Because of progressive hypoxemia, venovenous ECMO therapy was initiated 13 days after admission to our hospital (Figure 1A). Subsequently, nasopharyngeal swabs and tracheal aspirates tested negative for SARS-CoV-2. After tracheotomy and weaning, ECMO therapy and invasive assisted ventilation were no longer required, but the patient still needed intermittent renal replacement therapy (IRRT) (Figure 1A). 

During the course of the disease, the patient developed laboratory signs of liver injury during ECMO therapy before the clinical appearance of jaundice with elevated bilirubin levels, but sustained synthetic liver function reflected by the international normalized ratio (INR) and serum albumin measurements (Figure 1B–E). A diagnosis of SSC-CIP was confirmed by endoscopic retrograde cholangiopancreatography (ERCP), showing intraductal filling defects in the intrahepatic bile ducts due to biliary casts. In addition, the patient received drugs that have previously been associated with SSC, including amoxicillin-clavulanate, and ketamine sedation [27,28,29]. Plasma levels of bilirubin and ammonia gradually increased after that, with stable liver synthesis reflected by normal values of the international normalized ratio (INR) without substituting coagulation factors (Figure 1D,E).

**Figure 2:**
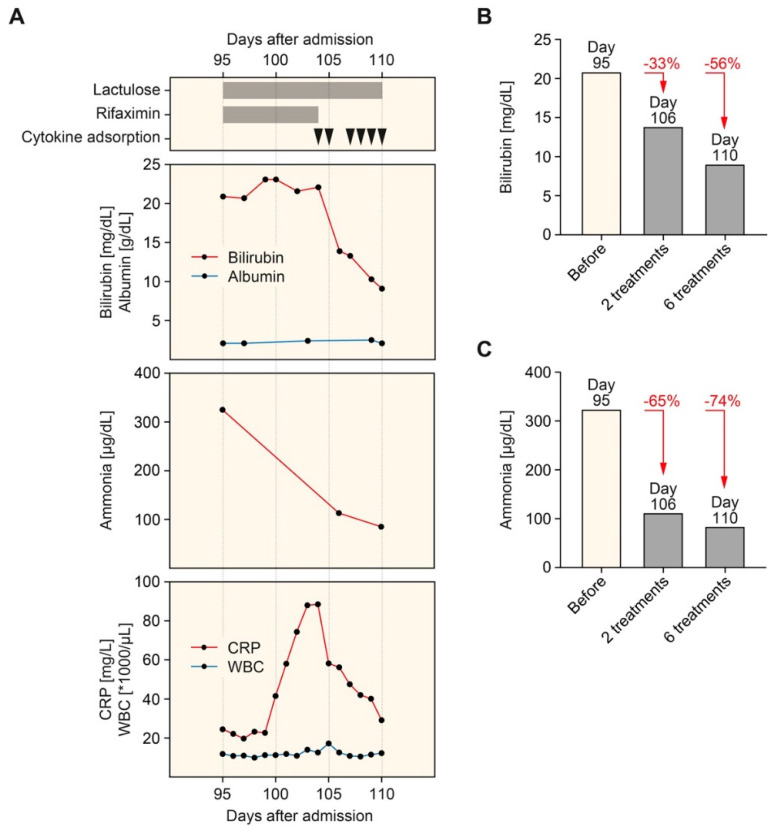
Timeline of hemadsorption therapy. (**A**) Arrowheads indicate the time points of hemadsorption. Plasma levels of bilirubin, albumin (upper panel), ammonia (middle panel), CRP, and WBC count (lower panel) are shown. (**B**) Levels of bilirubin before (95 days after admission) and after the initiation of hemadsorption therapy (106 and 110 days after admission). (**C**) Levels of ammonia before (95 days after admission) and after the initiation of hemadsorption therapy (106 and 110 days after admission). Abbreviations: CRP, C-reactive protein; WBC, white blood cell.

Nevertheless, the patient developed progressive nausea, vomiting, weakness, and exhaustion as the disease progressed. Hepatic encephalopathy was treated with lactulose and rifaximin, but clinical symptoms worsened (Figure 2A). Based on these observations, hemadsorption using the CytoSorb hemoperfusion device (CytoSorbents Europe, Berlin, Germany) was used in combination with IRRT (6 treatments within 7 days and 8–12 h per session). In this patient with SSC following ECMO therapy and severe COVID-19, hemadsorption successfully eliminated bilirubin, ammonia, and C-reactive protein (CRP) levels, while serum albumin levels remained stable (Figure 2A). Direct comparison revealed that bilirubin was less efficiently eliminated by hemadsorption by 33% and 56% after 2 and 6 treatments, respectively (Figure 2B) compared to ammonia by 65% and 74% after 2 and 6 treatments, respectively (Figure 2C). During successful elimination of bilirubin and ammonia, clinical symptoms of nausea, vomiting, weakness, and exhaustion improved. Following a continuation of IRRT but termination of hemadsorption therapy, bilirubin (32.2 mg/dL) and ammonia levels (208 g/dL) increased again, worsening clinical symptoms within 20 days. The patient suddenly died due to cardiac arrhythmia before liver transplantation evaluation was initiated.

## 3. Discussion

During acute liver dysfunction, a range of toxic metabolites accumulate in the blood. These metabolites include hydrophobic, albumin-bound molecules such as unconjugated bilirubin, bile acids, phenols, aromatic amino acids, and fatty acids, in addition to water-soluble compounds such as ammonia and circulating cytokines. Water-soluble metabolites, including ammonia, are in principle removed by hemodialysis but require high cut-off membranes for sufficient clearance [30,31,32,33,34,35]. In contrast, protein-bound liver toxins, including bilirubin, are not effectively removed by hemodialysis alone [36,37]. The aim of adsorption technologies as liver support system is the removal of such albumin-bound molecules. In in vitro studies, bilirubin is effectively removed by hemadsorption with a minimal loss of albumin itself [38,39]. Therefore, combining hemadsorption with hemodialysis is an attractive approach for eliminating protein-bound and water-soluble metabolites in patients with severe liver dysfunction. Various blood purification systems are available as liver support therapies to eliminate toxic metabolites in acute liver dysfunction [39]. In particular, methods of albumin dialysis have been frequently used for the clearance of protein-bound liver toxins [19,20]. Beside this, these systems are complicated to use, expensive, and have limited value in daily intensive care practice [21,22]. In addition, a survival benefit has not been demonstrated in randomized clinical trials [40,41]. However, there is evidence that the use of liver support systems improves clinical symptoms of liver dysfunction and might reduce mortality not attributed to a particular liver support system [13].

The application of hemadsorption is best studied in the context of sepsis. The inflammatory state associated with sepsis leads to proinflammatory cytokine release into the systemic circulation with deleterious effects and high mortality [42,43,44]. These observations resulted in efforts to attenuate this inflammatory response by extracorporeal cytokine removal using CRRT [45]. However, no randomized controlled trials could demonstrate a survival benefit of using CRRT for cytokine removal, including the use of high-volume procedures [46,47,48]. In contrast to CRRT, multiple experimental studies using animal models of sepsis have demonstrated the efficacy of hemadsorption for reducing various circulating cytokines and chemokines (including CRP) associated with attenuation of organ injury and improved survival [49,50,51,52,53]. According to the manufacturer’s data, hemadsorbers have a surface area of about 45,000 m^2^ and eliminate molecules up to 55 kDa in size. In contrast to soluble cytokines and chemokines in circulation, most liver toxins are bound to plasma albumin (39). Protein-bound bilirubin and most bile acids are below 55 kDa in size and can, therefore, principally be eliminated through hemadsorption [26,39]. However, water-soluble metabolites, including ammonia with 17 kDa, are only removed by high-flux hemodialysis [30,31,32,33,34,35]. While the effectiveness of additional hemadsorption to eliminate protein-bound bilirubin and water-soluble ammonia has been described previously in critically ill patients with acute liver failure, treatment efficacy in SSC-CIP has not yet been reported [54,55,56]. The pathogenesis of SSC-CIP remains elusive, but current concepts suggest that the primary insult is hypoperfusion of the biliary vasculature. The intrahepatic cholangiocyte epithelium is supplied by the peribiliary vascular plexus that arises from the hepatic arteries. This contrasts with the liver parenchyma supplied by a dual blood source arising from the hepatic arteries and portal system. Therefore, the biliary vasculature is thought to be more susceptible to hypoperfusion and consecutive ischemia [57]. SSC-CIP has previously been observed after ECMO therapy, including in cases of COVID-19, and a direct viral tropism with detection of SARS-CoV-2 RNA and associated nucleocapsid protein in cholangiocytes and bile ducts has been proposed [58,59]. Finally, endotheliitis resulting in hypercoagulability in the peribiliary vascular plexus may also aggravate ischemia of the biliary tract.

We herein expand current knowledge and describe a case of SSC-CIP following ECMO therapy and severe COVID-19 treated by hemadsorption. The main limitation is a case description, and further clinical research is required to strengthen our observations. Moreover, hemadsorption has already been described in the context of acute liver failure [54,55,56]. This is particularly relevant since it has been reported that about one-third of patients critically ill with COVID-19 suffer from liver dysfunction associated with high mortality [60]. Moreover, this is the first report of SSC-CIP treated with hemadsorption, especially relevant since SSC-CIP affects a considerable number of COVID-19 patients requiring ECMO therapy [58]. By combining hemadsorption with hemodialysis treatment, successful bilirubin and ammonia elimination were observed. More importantly, our patient showed that extracorporeal clearance of bilirubin and ammonia were associated with improved clinical symptoms, including nausea, vomiting, weakness, and exhaustion.

## 4. Conclusions

In conclusion, the kinetics of bilirubin and ammonia during hemadsorption therapy confirmed efficient removal in this case of SSC following ECMO therapy and severe COVID-19, associated with regredient clinical symptoms of chronic liver dysfunction. Furthermore, direct comparison revealed that ammonia is more efficiently eliminated by hemadsorption than bilirubin levels.

## Data Availability

Deidentified data are available upon reasonable request from the corresponding author.
